# Supplementary material for: A whole-animal phenotypic drug screen identifies suppressors of atherogenic lipoproteins
Source: eLife. 2026 Aug 27;14:RP105314. doi: 10.7554/eLife.105314 (PMC13521566; doi:10.7554/eLife.105314)
Supplement: Supplementary file 2 [file elife-105314-code1.zip › Output_data/rdu14_nPAGE.pdf]

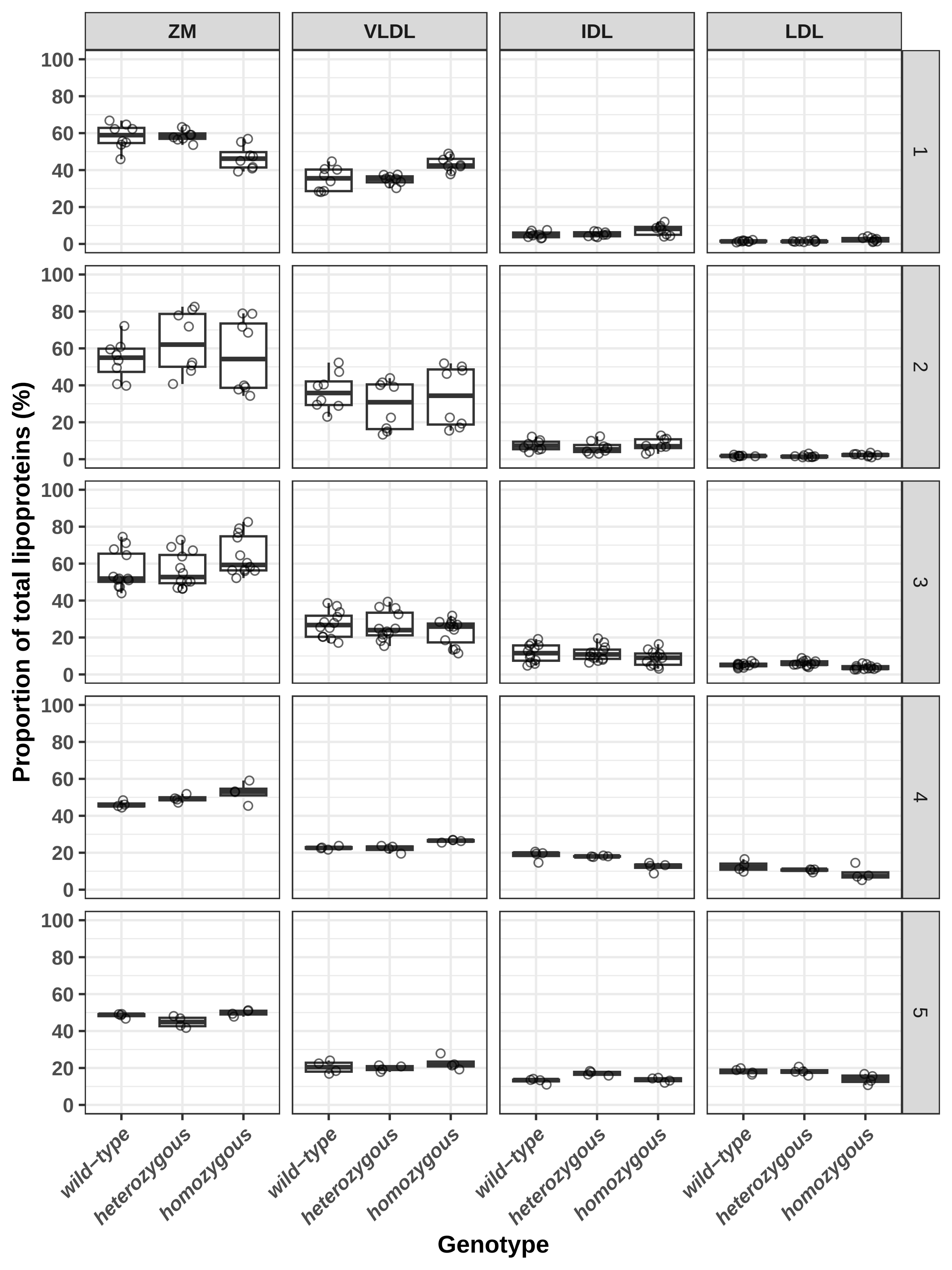

ZM
VLDL
IDL
LDL
1
2
3
4
5
100
80
60
40
20
0
Proportion of total lipoproteins (%)
wild−type
heterozygous
homozygous
Genotype
